# Structural Investigations on the SH3b Domains of *Clostridium perfringens* Autolysin through NMR Spectroscopy and Structure Simulation Enlighten the Cell Wall Binding Function

**DOI:** 10.3390/molecules26185716

**Published:** 2021-09-21

**Authors:** Yubao Shan, Xiaoling He, Zi Wang, Xiali Yue, Jiang Zhu, Yunhuang Yang, Maili Liu

**Affiliations:** 1 Department of Chemistry, College of Science, Huazhong Agricultural University, Wuhan 430070, China; shanyubao@wipm.ac.cn; 2 State Key Laboratory of Magnetic Resonance and Atomic Molecular Physics, Key Laboratory of Magnetic Resonance in Biological Systems, National Center for Magnetic Resonance in Wuhan, Wuhan Institute of Physics and Mathematics, Innovation Academy for Precision Measurement Science and Technology, Chinese Academy of Sciences—Wuhan National Laboratory for Optoelectronics, Wuhan 430071, China; hexl@apm.ac.cn (X.H.); wangzi@wipm.ac.cn (Z.W.); yang_yh@apm.ac.cn (Y.Y.); ml.liu@apm.ac.cn (M.L.); 3 University of Chinese Academy of Sciences, Beijing 100049, China

**Keywords:** Acp, SH3b, NMR structure, peptidoglycan hydrolase, cell wall

## Abstract

*Clostridium perfringens* autolysin (CpAcp) is a peptidoglycan hydrolase associated with cell separation, division, and growth. It consists of a signal peptide, ten SH3b domains, and a catalytic domain. The structure and function mechanisms of the ten SH3bs related to cell wall peptidoglycan binding remain unclear. Here, the structures of CpAcp SH3bs were studied through NMR spectroscopy and structural simulation. The NMR structure of SH3b6 was determined at first, which adopts a typical β-barrel fold and has three potential ligand-binding pockets. The largest pocket containing eight conserved residues was suggested to bind with peptide ligand in a novel model. The structures of the other nine SH3bs were subsequently predicted to have a fold similar to SH3b6. Their ligand pockets are largely similar to those of SH3b6, although with varied size and morphology, except that SH3b1/2 display a third pocket markedly different from those in other SH3bs. Thus, it was supposed that SH3b3-10 possess similar ligand-binding ability, while SH3b1/2 have a different specificity and additional binding site for ligand. As an entirety, ten SH3bs confer a capacity for alternatively binding to various peptidoglycan sites in the cell wall. This study presents an initial insight into the structure and potential function of CpAcp SH3bs.

## 1. Introduction

Gram-positive bacteria develop a thick cell wall which is a mesh polymer of peptidoglycans to protect the cells. Peptidoglycan consists of glycan chains that are cross-linked with peptide side chains [1]. A variety of lytic enzymes can hydrolyze peptidoglycans of the cell wall to regulate bacteria growth or infection [2]. These peptidoglycan hydrolases usually have a cell-wall-binding (CWB) module recognizing the peptide side chains and a catalytic domain cleaving specific type of linkage in peptidoglycan. According to the cleaving sites, they can be divided into different classes such as glucosaminidase, muramidase, amidase, and endopeptidase [3]. Due to their role in degrading bacteria cell wall, peptidoglycan hydrolases are increasingly engineered as alternatives to antibiotics against bacteria [4,5].

**Figure 1:**
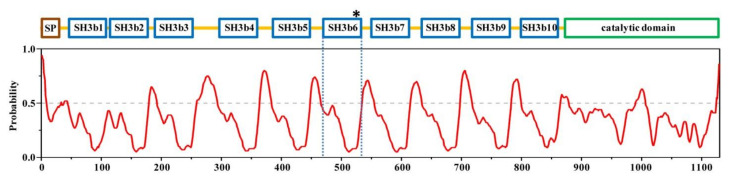
Architecture of CpAcp. Schematic domain structure of CpAcp with an analysis of structurally disordered probability at bottom. SP, signal peptide; SH3b, bacterial Src homology 3. The regions showing a probability above 0.5 (marked with a gray dashed line) are considered to be disordered. SH3b6 is highlighted with asterisk and blue dashed line.

*Clostridium perfringens* is a Gram-positive, rod-shaped, spore-forming, and anaerobic bacterium that can be found on raw meat, in the intestines of animals, and in the environment. *C. perfringens* bacteria are one of the most common causes of foodborne illnesses such as food poisoning [6]. *C. perfringens* autolysin (CpAcp, UniProt ID: Q8XL11) encoded by gene *CPE1231* is a peptidoglycan hydrolase expressed at growth phase and sporophore formative phase, and is associated with cell separation, division, and growth [2,7]. CpAcp has 1129 amino-acid residues and consists of a signal peptide, a CWB module containing ten tandem bacterial Src homology 3 (SH3b) domains, and a catalytic domain belonging to the N-acetylglucosaminidase family (Figure 1) [7,8]. The crystal structure of CpAcp catalytic domain has been determined to adopt a crescent-shaped fold with a deep groove at the center for substrate binding [8]. However, the structures of the ten SH3b domains of CpAcp have not been reported so far.

The SH3b domains in the CWB module of peptidoglycan hydrolases are considered to be essential for cell wall recognition and binding [9,10,11]. Many SH3b structures from various peptidoglycan hydrolases have been determined either in the isolated state or along with the catalytic domain, such as lysostaphin from *Staphylococcus simulans* [12], lysostaphin homolog ALE-1 from *Staphylococcus capitis* [10], Atl from *Staphylococcus epidermidis* [13], LytB from *Streptococcus pneumonia* [14], etc. These SH3b structures share a similar β-barrel fold consisting of 6~8 β-strands that can be roughly divided into two sheets, which is comparable to the well-known eukaryotic SH3 domains that target proline-rich peptides [15]. On the other hand, SH3bs show low sequence similarity to eukaryotic SH3s, suggesting that their functions are different to some extent [16]. Consistent with this, several studies have evidenced that SH3b domains of lysostaphin and ALE-1 recognize the interpeptide pentaglycine cross bridge in peptidoglycan [10,12,17]. However, not only the sequence of different SH3bs but also the number of tandem SH3bs in different peptidoglycan hydrolases varied considerably, suggesting the diverse binding specificity of the CWB modules of peptidoglycan hydrolases.

In this study, the solution structure of CpAcp SH3b6 was determined using NMR spectroscopy. Based on the structure, the function of SH3b6 was predicted through morphology analysis of the potential ligand-binding pockets and structure similarity comparison with the SH3b homologues having known ligands. Molecular docking implied a novel binding model of SH3b6 for peptide ligand. Meanwhile, the structural models of SH3b1/2/3/10 of CpAcp were built through homologous modeling, by which the overall cell-wall-binding function of CpAcp was supposed. This study will facilitate the comprehensive understanding of the function mechanism of CpAcp, and will guide the further experimental investigation on the binding of CpAcp SH3bs with peptide ligand from the cell wall peptidoglycan, which is beyond the scope of this article.

## 2. Results and Discussion

### 2.1. Structural Architecture of CpAcp

*C. perfringens* Acp is a large protein divided into at least 12 domains with different functions as aforementioned. To have an overall look into the structural architecture of CpAcp, PrDOS [18] (http://prdos.hgc.jp/cgi-bin/top.cgi) was used to analyze the disordered/ordered probability of full-length CpAcp. The result showed that all previously identified domains, including signal peptide, SH3bs, and catalytic domain, fell within ordered regions (disordered probability < 0.5), while the interdomain linkers were most disordered, except the short linker (5-residue long) between SH3b1 and SH3b2 (Figure 1). The lengths of interdomain linkers of CpAcp are mostly within the scope of 15–21 residues, whereas there are two linkers out of this range, which are the short SH3b1/2 linker mentioned above and the extremely long SH3b3/4 linker consisting of 41 residues. The long disordered interdomain linkers over 15 residues can usually confer flexibility facilitating the relatively independent motion of domains [19]. Thus, the SH3bs of CpAcp except SH3b1/2 were expected to motion independently and function individually, although interdomain regulation through interaction cannot be excluded. On the other hand, due to the short linker, SH3b1/2 may function synergistically.

### 2.2. NMR Structure of CpAcp SH3b6

**Figure 2:**
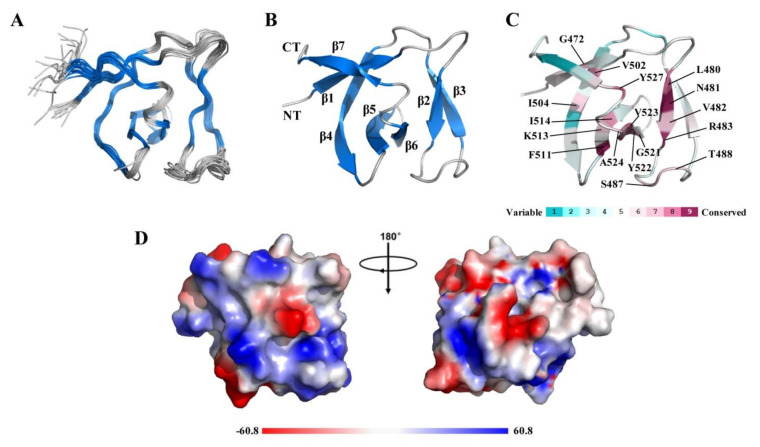
Solution NMR structure of CpAcp SH3b6. (**A**) Superposition of the backbone traces of 20 conformers of CpAcp SH3b6 with lowest energy. The β-strands are colored in marine blue and the loops are in gray. (**B**) The cartoon representation of CpAcp SH3b6 structure with β-strands labeled. CT, C-terminus; NT, N-terminus. (**C**) ConSurf analysis with the nine-color conservation scores projected onto CpAcp SH3b6 structure with the lowest energy. Residues with conservation score over 7 are indicated. (**D**) Electrostatic surface potential diagrams of CpAcp SH3b6 colored by electrostatic potential.

In order to perform a detailed investigation on the structure of CpAcp CWB module, solution structure of SH3b6 (E469-V535) was determined using NMR spectroscopy at first (Figure 2A). The RMSD values for backbone atoms and heavy atoms in the ordered regions were 0.3 and 0.9 Å, respectively (Table 1, PDB ID: 2KT8). The overall structure adopted a β-barrel fold consisting of 7 β-strands (β1, 470–474; β2, 480–483; β3, 492–496; β4, 501–508; β5, 511–516; β6, 519–524; β7, 528–532) (Figure 2B). The β2 and β3 strands formed a β-hairpin that is exclusively found in SH3b but not in eukaryotic SH3 domains, while the other 5 β-strands are usually present in both SH3b and eukaryotic SH3 domains. The region of CpAcp SH3b6 containing the β2 and β3 strands corresponded to the well-known RT loop of eukaryotic SH3, which is usually engaged in binding to proline-rich peptides [15].

ConSurf [20] analysis using 300 homologous sequences was performed for SH3b6 to identify evolutionarily conserved residues. As shown in Figure 2C, 17 residues with conserved score over 7 were found, among which S487, T488, and Y527 were located in loops, while the other 14 residues were located in β-strands, especially β2/5/6. Electrostatic surface potential analysis did not uncover particular regions lumped with positively or negatively charged residues (Figure 2D), implying that SH3b6 may not bind with highly charged ligand. Taken together, CpAcp SH3b6 forms a typical SH3b structure potential for binding to cell wall peptidoglycan.

### 2.3. Potential Ligand-Binding Pocket of CpAcp SH3b6

**Figure 3:**
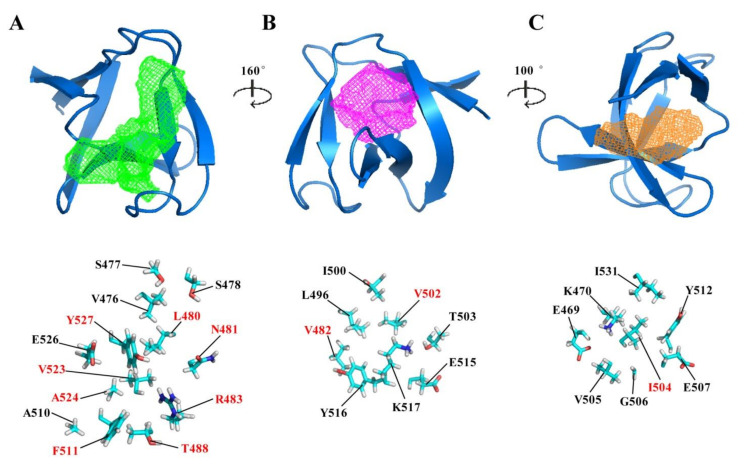
Potential ligand-binding pockets of CpAcp SH3b6. (**A**–**C**) Three pockets identified using online software DoGSiteScorer are shown with mesh and arranged according to their size in green, magenta, and orange color, respectively. The residues with side chains involved in forming the respective pockets are shown as sticks in the images at bottom. The residues labeled with red font are those with conserved score over 7 identified in ConSurf analysis.

Eukaryotic SH3 domains usually contain a cleft or 2~3 adjacent pockets for binding to proline-rich peptide [15]. Correspondingly, SH3b domains such as those in lysostaphin and ALE-1 have the cleft or pockets for binding to pentaglycine peptide [12,17]. Moreover, there are also pockets in SH3b domains probably binding to small molecules as found in several SH3b crystal structures [14,21,22,23]. Based on the CpAcp SH3b6 structure, the potential ligand-binding pockets were identified using online software DoGSiteScorer [24] (https://proteins.plus/#dogsite). The result uncovered three pockets with different sizes of CpAcp SH3b6 (Figure 3 and Table 2), termed hereafter as P1, P2, and P3 from the largest to the smallest. All three pockets contained almost equal ratios of nonpolar and polar/charged residues, suggesting an amphipathic binding capacity. P1 was a long groove with a volume of 317.38 Å^3^, formed by β1-β2 loop, β2, β2-β3 loop, β5, β6, and β6-β7 loop. It is worth noting that eight highly conserved residues were involved in P1 (Figure 3A), implying a conserved function of P1. P2 had a volume of 210.82 Å^3^, formed by β2, β3, β3-β4 loop, β4, β5, and β6, while P3 had a volume of 125.25 Å^3^, formed by β1, β4, β5, and β7. The residues in P2 and P3 were significantly less conserved than those in P1 (Figure 3B,C), suggesting that their functions may be variable among homologues.

### 2.4. Structural Comparison of CpAcp SH3b6 with Other SH3b Proteins

**Figure 4:**
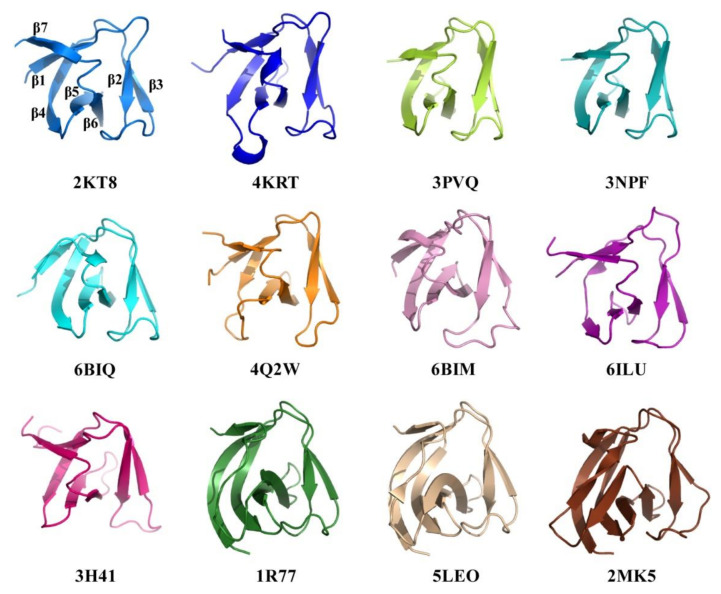
Structure similarity of CpAcp SH3b6 with its SH3b homologues. The structures of CpAcp SH3b6 (2KT8) and its homologues with structural similarity identified using Dali server are shown with cartoon representations in different colors, and the respective PDB IDs of the structures are indicated at bottom. The secondary structure elements of CpAcp SH3b6 are labeled for comparison.

Structure similarity analysis of CpAcp SH3b6 was carried out using Dali [25] server, and 100 peptide chains with Z-scores over 6.0 were identified. Most of these homologues showed sequence identity lower than 30% with CpAcp SH3b6. Those having been reported by published papers are summarized in Table 3 and partly shown in parallel with CpAcp SH3b6 in Figure 4. The listed 14 structures belong to 11 SH3b-containing peptidoglycan hydrolases. The structures 5LEO, 5NMY, 6RK4, and 6RJE belonging to *S. simulans* lysostaphin exhibit the same fold as SH3b. The sequence identities of these 11 SH3bs compared to CpAcp SH3b6 range from 27% to 10%. Overall structures of CpAcp SH3b6 and the 11 SH3b homologues share a similar β-barrel fold, but local conformations are diverse to various extents. The structures 4KRT, 3PVQ, 3NPF, 6BIQ, 4Q2W, 6BIM, 6ILU, and 3H41 are largely similar to CpAcp SH3b6, although modest differences can be found individually. β4-β5 loop of 4KRT is very long and adopts a short helix. β7 of 6BIQ turns out to be a rigid loop. β4 of 4Q2W is short. β4 of 6ILU adopts two short separated strands and β5 and β6 are shorter. β2-β3 loop of 3H41 adopts an inverse conformation compared to CpAcp SH3b6. Nevertheless, remarkable differences of 1R77, 5LEO, and 2MK5 with the above nine structures can be found as long β1 and a sequence insertion in the region corresponding to β6-β7 loop of CpAcp SH3b6. The inserted sequence adopts a loop in 1R77, a β-strand in 5LEO, and two β-strands in 2MK5, respectively.

**Figure 5:**
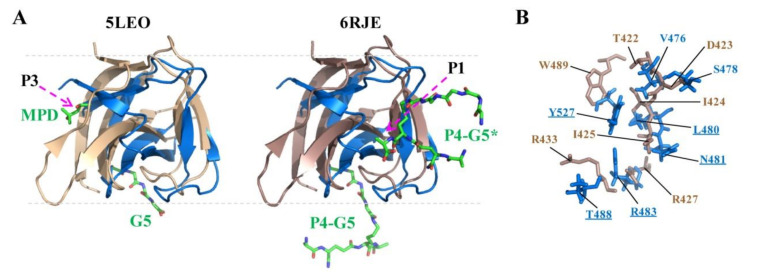
Structure comparison of CpAcp SH3b6 with lysostaphin SH3b. (**A**) Superposition of the structure of CpAcp SH3b6 (marine) with those of lysostaphin SH3b bound by different ligands with PDB IDs of 5LEO (wheat) and 4RJE (dark salmon). The bound ligands are shown as sticks and labeled with green font. MPD, (4S)-2-methyl-2,4-pentanediol; G5, pentaglycine; P4–G5 and P4–G5*, AγQK[GGGGG]A peptide. The positions for P1 and P3 pockets of CpAcp SH3b6 are marked with dashed arrows. (**B**) The residues in the binding site of lysostaphin SH3b for P4–G5* and the corresponding residues of CpAcp SH3b6 P1 are shown as sticks and colored in dark salmon and marine, respectively.

Among the listed structures, 5LEO [12] and 6RK4/6RJE [17] represent *S. simulans* lysostaphin SH3b complexed with pentaglycine cross-bridge peptide (G5) and a tetrapeptide stem with the pentaglycine cross bridge as a lateral chain (AγQK[GGGGG]A; P4–G5; K5T), respectively. The CpAcp SH3b6 structure was superimposed with 5LEO and 6RJE to compare the ligand-binding pockets (Figure 5A). In both 5LEO and 6RJE, lysostaphin SH3b utilizes a binding site for G5 that was not detected in CpAcp SH3b6, indicating that CpAcp SH3b6 has a different binding model for G5. On the other hand, in 6RJE, lysostaphin SH3b utilizes a second binding site for the tetrapeptide stem (AγQKA), which is partly overlapped with the P1 pocket of CpAcp SH3b6. In particular, the pocket residues of lysostaphin SH3b for tetrapeptide stem show similar conformation and nonpolar/polar property with the P1 pocket residues of CpAcp SH3b6 (Figure 5B), supporting the peptide-binding potential of P1. In addition, a (4S)-2-methyl-2,4-pentanediol (MPD) molecule was found to bind to a third site of lysostaphin SH3b corresponding to the P3 pocket of CpAcp SH3b6. However, the structures for this region between lysostaphin SH3b and CpAcp SH3b6 are quite different, implying that CpAcp SH3b6 P3 may bind to a different small molecule. Taken together, the P1 pocket of CpAcp SH3b6 can be expected to serve as a binding pocket for peptide ligand.

### 2.5. Docking of CpAcp SH3b6 with P4-G5 Peptide

**Figure 6:**
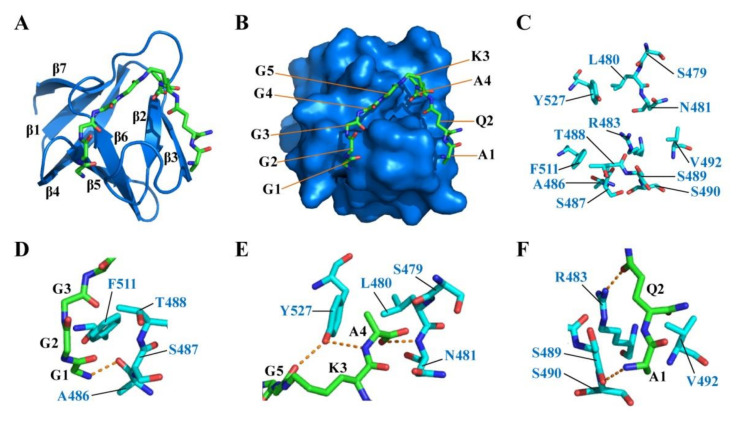
Binding model of CpAcp SH3b6 with P4-G5 (K5T) peptide. (**A**) Complex model of CpAcp SH3b6 bound by P4-G5 with SH3b6 shown in cartoon (marine) and P4-G5 peptide in stick representation. Secondary elements of SH3b6 are marked. (**B**) Complex model of CpAcp SH3b6 bound by P4-G5 with SH3b6 in surface (marine) representation. Amino-acid units of P4-G5 are marked. (**C**) The residues of SH3b6 involved in interaction with P4-G5 are shown as sticks. (**D**–**F**) Three interacting sites between SH3b6 and P4-G5 are shown with carbon colored in cyan and green, respectively. The interacting residues are indicated. Hydrogen bonds are shown as orange dashed lines.

In order to support the speculation that P1 pocket of CpAcp SH3b6 can bind with peptide ligand, the peptide P4-G5 (K5T) that represents a repeat unit in cell wall peptidoglycan was used to dock with CpAcp SH3b6 structure. The result showed that the G1–G5, K3, and A4 residues of P4-G5 were docked to the P1 pocket of SH3b6, while A1 and Q2 were bound to a region next to P1 (Figure 6A). P4-G5 adopted an arc conformation extending along the surface of SH3b6, with A4 inserting in a cavity (Figure 6B). Twelve residues of SH3b6 were involved in interaction with P4-G5 (Figure 6C).

The interaction interface can be generally divided into three regions: G1–G3 residues were bound by A486, S487, T488, and F511 (Figure 6D); G5, K3, and A4 were bound by S479, L480, N481, and Y527 (Figure 6E); A1 and Q2 were bound by R483, S489, S490, and V492 (Figure 6F). L480, T488, V492, F511, and Y527 conferred hydrophobic interactions with the peptide backbone. Six hydrogen bonds were found between residue pairs including G1-A486, G5-Y527, A4-Y527, A4-N481, Q2-R483, and A1-S489. Importantly, side-chains of R483 and Y527 were involved in forming hydrogen bonds, suggesting that the two residues may be related to recognition specificity. The docking result strongly supports the hypothesis that the P1-pocket-involved region of SH3b6 can function in peptide ligand binding, and implies a novel binding model different from the known model of lysostaphin SH3b (Figure 5A).

### 2.6. Structure Simulation of CpAcp SH3b1/2/3/10

**Figure 7:**
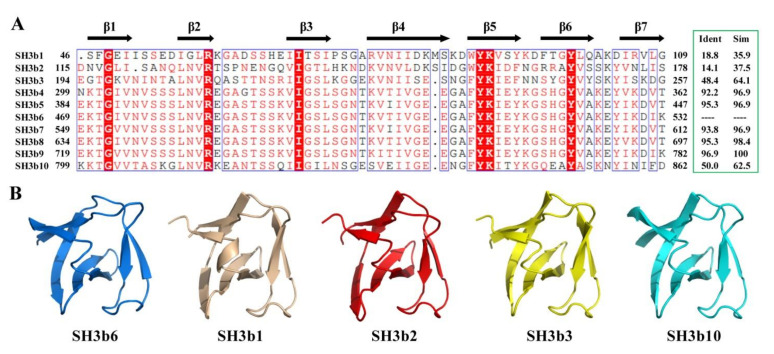
Sequence and structure of CpAcp SH3bs. (**A**) Sequence alignment of 10 SH3bs of CpAcp rendered using ESPript [31] with default settings for similarity calculations. Identical (white letters filled with red color) and similar (red letters with blue box) amino acids are denoted. Secondary structural elements of SH3b6 are indicated above the amino acid sequence. The identity and similarity of SH3b sequences compared to SH3b6 are shown in the green box. (**B**) Structure models of SH3b1/2/3/10 and NMR structure of SH3b6 are shown in the cartoon with individual color.

The determination of CpAcp SH3b6 domain structure encouraged us to predict the structures of the other nine SH3b domains. Before doing this, sequence identity and similarity of the 10 SH3b domains were analyzed (Figure 7A). The result showed that the sequences of SH3b4/5/7/8/9 are highly similar (over 95%) to SH3b6. SH3b3/10 show moderate similarity (64.1% and 62.5%), while SH3b1/2 display low similarity (35.9% and 37.5%) to SH3b6. SH3b1/2 have moderate sequence identity and similarity of 20.3% and 53.1% to each other.

Subsequently, the structures of the nine CpAcp SH3bs were simulated with the I-TASSER [30] server. The resulted structures of SH3b4/5/7/8/9 were identical to the SH3b6 structure, and thus are not shown. The predicted SH3b1/2/3/10 structures were also very similar to SH3b6 structure (Figure 7B). Differences in secondary structure element were mainly found for β4 and β7. β4 is longer in SH3b3/10 compared to SH3b6, while is separated into two strands in SH3b1/2. β7 is shorter in SH3b1/2/3 than SH3b6. Generally, the 10 SH3b structures of CpAcp are very similar.

**Figure 8:**
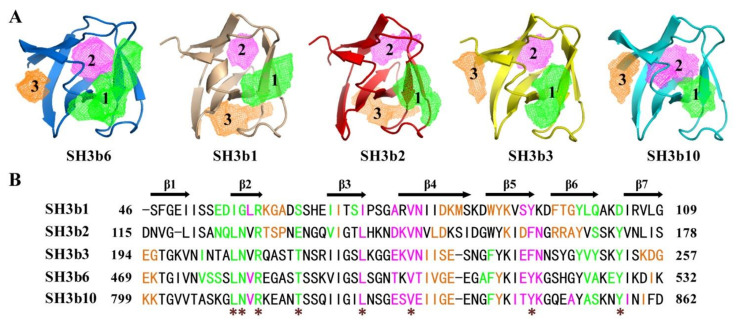
Potential ligand-binding pockets of CpAcp SH3b1/2/3/10. (**A**) Three pockets were identified for respective structure models of SH3b1/2/3/10 and are shown with mesh. The pockets are numbered according to those of SH3b6 and respectively colored with green, magenta, and orange. (**B**) Sequence alignment of SH3b1/2/3/6/10 with the residues involved in forming individual pockets colored in the same manner in (**A**). Secondary structural elements of SH3b6 are indicated above the amino acid sequence. Conserved pocket residues among the five SH3bs are marked with asterisks.

Based on the structure models, the potential pockets of SH3b1/2/3/10 were analyzed. Similar location of P1 and P2 pockets to SH3b6 were found for all four SH3bs, although the size and morphology varied a lot (Figure 8A and Table 4). SH3b3/10 showed a similar P3 pocket location to SH3b6. However, SH3b1/2 did not have a similar P3 pocket with SH3b6. Instead, they showed a pocket at the location similar to that found in lysostaphin SH3b for binding with G5. Four pockets were identified for lysostaphin SH3b (Pa-Pd), previously [12]. Pa (volume 273.0 Å^3^) is for binding to pentaglycine, Pb (194.1 Å^3^) for MPD, and Pc (158.3 Å^3^) for tetrapeptide stem. P1 of CpAcp SH3b1/2 corresponds to Pc of lysostaphin SH3b in location, while P3 corresponds to Pa. However, the sizes of these pockets are not consistent. In particular, the secondary elements and residues involved in CpAcp SH3b1/2 P3 formation vary a lot compared to lysostaphin SH3b Pa. Thus, the function of P3 of SH3b1/2 remains ambiguous.

The differently engaged residues of the pockets of SH3b1/2/3/6/10 should contribute to the varied location and morphology of the pockets (Figure 8B). In common, the residues in β1-β2 loop, β2, β2-β3 loop, β6, and β6-β7 loop formed P1 pockets, while those in β3, β3-β4 loop, β4, and β5 formed P2 pockets for all five structures. Eight conserved residues were found to be involved in forming P1 and P2 pockets for all five structures (Figure 8B, asterisk labeled). Therein, the five residues in P1 have been highlighted in comparison with lysostaphin SH3b in Figure 5B (underlined), and showed contacts with P4-G5 peptide in the complex model (Figure 6). However, residues in β3 were also involved in P1 of SH3b1/2, instead of the residue in β5 of SH3b3/6/10 P1, suggesting a slight difference. Moreover, the residues in β1, β4, and β7 formed P3 of SH3b3/6/10, and those in β2-β3 loop, β3, β4, β5, and β6 formed P3 of SH3b1/2, indicating a significantly different P3 composition. Taken together, SH3b3-10 should have a similar ligand-binding capacity, while SH3b1/2 may have a different specificity and additional binding site for ligand. As an entirety, 10 SH3bs of CpAcp were expected to cooperatively target complex cell wall compositions and achieve binding ability to extensive sites. Meanwhile, considering the similar ligand-binding ability of SH3b3-10 and the disordered/flexible interdomain linkers, we hypothesize that through dynamically alternating the SH3bs bound with the peptide bridge or stem, CpAcp can rapidly shift along peptidoglycan network surface like a “wiggling worm” for substrate cleaving.

## 3. Materials and Methods

### 3.1. Protein Expression and Purification

The coding gene of CpAcp/CPE1231 SH3b6 domain (E469-V535) was cloned into an NESG-modified pET21 expression vector including a C-terminal 6×His-tag (LEHHHHHH). The recombinant plasmid was transformed into *Escherichia coli* BL21 (DE3) cells for protein expression. *U*-^13^C, ^15^N (NC), and *U*-^15^N, 5% biosynthetically directed ^13^C (NC5) samples of SH3b6 were expressed and purified following a standard protocol described previously [32]. Briefly, the *E. coli* cells containing the above plasmid were grown in MJ minimal medium at 37 °C until induction with a final concentration of 0.5 mM isopropyl-β-d-thiogalactopyranoside (IPTG) at an OD_600_ of ~0.7, followed by culturing overnight at 20 °C for 16 h. The cells were harvested by centrifugation and lysed by sonication. The lysate was clarified by centrifugation at 20,000 rpm for 30 min and filtration. The supernatant was purified using the ÄKTAxpress^TM^ chromatography system (GE Healthcare) with a Ni-affinity column (HisTrap IMAC HP^TM^ column, 5 mL) followed by a gel filtration column (HiLoad 26/60 Superdex 75pg). The purified protein was concentrated to a final concentration of 1.0 mM for study in the NMR buffer containing 20 mM NH_4_OAc, 100 mM NaCl, 5 mM CaCl_2_, 10 mM DTT, 0.02% NaN_3_, and 90% H_2_O/10% D_2_O (*v*/*v*) at pH = 4.5.

### 3.2. Chemical Shift Assignments

All NMR experiments were carried out at 298 K on a Varian Inova 600 MHz spectrometer equipped with a 5 mm HCN cryogenic probe and a Bruker Avance III 850 MHz spectrometer equipped with a 5 mm HCN conventional room temperature probe. The collected NMR data included: 2D ^1^H-^15^N HSQC, ^1^H-^13^C HSQC, 3D HNCO, HN(CA)CO, HNCA, HN(CO)CA, HNCACB, CBCA(CO)NH, HBHA(CO)NH, H(CCO)NH, and C(CO)NH for backbone assignments, and 2D ^1^H-^13^C HSQC (aliphatic and aromatic), 3D H(C)CH-TOCSY, H(C)CH-COSY, (H)CCH-TOCSY, and 4D CC NOESY for side chain assignments. The stereospecific assignments of isopropyl methyl groups of Val and Leu residues were determined from 2D constant-time ^1^H-^13^C HSQC spectrum for the NC5 sample [33]. The data were processed using the NMRPipe [34] and analyzed with the Sparky program [35]. The backbone and side-chain resonances were automatically assigned using the PINE server from NMRFAM [36], and subsequently validated and corrected manually. All assignments were further confirmed from NOESY spectra, including 3D ^15^N-edited NOESY-HSQC and ^13^C-edited NOESY-HSQC. Chemical shift assignments have been deposited in the BioMagResBank (BMRB accession number 16688).

### 3.3. NMR Structure Calculation

NOE-based interproton distance constraints were determined automatically for CpAcp SH3b6 using CYANA 3.0 [37]. Chemical shift assignments, NOESY peak lists from four NOESY spectra with peak intensities, and the constraints for backbone phi (ϕ) and psi (ψ) torsion angle derived from chemical shifts of backbone atoms using the TALOS+ software program [38] were used as input for CYANA 3.0. Manual and iterative refinement of the NOESY peak selection list was performed using RPF NMR quality assessment to calculate the “fit” between the structure and the NOESY peak list [39]. During the iterations near the end of the structure calculations, hydrogen bonding constraints on NH and CO distances were introduced based on the proximity of potential donors and acceptors identified in the initial structure. Through the explicit hydrodynamic fields of CNS 1.2 and PARAM19, 20 of the 100 structures calculated in CYANA 3.0 were refined with minimal energy using NOE-derived final distance constraints, hydrogen bond constraints, and TALOS-derived dihedral angle constraints. The final NMR ensemble of 20 structures and all restraints used in the structure calculations have been deposited in the Protein Data Bank (PDB ID 2KT8). Structural statistics and overall structural quality factors were calculated using PSVS [40] version 1.4 (Table 1).

### 3.4. Structure Modeling

The structure models of SH3b1/2/3/4/5/7/8/9/10 were built through de novo homology modeling using I-TASSER (http://zhanglab.ccmb.med.umich.edu/I-TASSER/) that first identifies structural templates from the PDB by the multiple threading approach, LOMETS, with atomic models constructed by iterative template-based fragment assembly simulations. The sequence of individual SH3b was entered into I-TASSER as input and then I-TASSER was run to simulate the structures. The details and descriptions can be referred to the manual. The first model (with the highest score) from resulting models for each input SH3b was chosen for further analysis.

The complex model of P4-G5 (K5T) and CpAcp SH3b6 was simulated by HDOCK [41], a protein-protein docking based on a hybrid algorithm of template-based modeling and ab initio free docking (http://hdock.phys.hust.edu.cn/). The PDB file of K5T (download from PDB database) and PDB ID of SH3b6 (2KT8) were respectively used as receptor and ligand and inputted into the HDOCK server guided by the manual. The resulting docking model of K5T and SH3b6 with the highest score (the top model) was chosen as the complex to be analyzed in this study.

## 4. Conclusions

Through NMR structure determination, molecular docking, homologous modeling, and a series of bioinformatic analysis, the structure and function of CpAcp CWB module consisting of 10 SH3bs were investigated preliminarily in this study. The NMR structure of CpAcp SH3b6 was determined to adopt a β-barrel fold with seven β-strands, similar to its SH3b homologues. Potential ligand-binding pocket analysis identified three pockets of CpAcp SH3b6, among which P1 with the largest size and eight conserved residues may function in binding to P4-G5 peptide in a novel model, while P2 and P3 with much smaller size and variable residues may bind with small molecules. In the CWB module, SH3b3-10 have similar structures and therefore similar potential ligand-binding pockets like SH3b6, while SH3b1/2 have similar structures but different P3 pockets from SH3b6, which is surprisingly comparable to the pocket of lysostaphin SH3b for pentaglycine cross bridge. Thus, SH3b3-10 should have similar ligand-binding ability, while SH3b1/2 may have different specificity and an additional binding site for ligand. Facilitated by the flexible interdomain linkers, the 10 SH3bs can together anchor CpAcp to the cell wall network, and provide an extensive binding specificity for different cell wall sites and structurally dynamic binding to move along the peptidoglycan network for target searching. This study presents a first insight into the structure and potential function of CpAcp SH3bs, will guide the further experimental investigation on the binding of CpAcp to cell wall compositions, and thereby facilitate the comprehensive understanding of the function mechanism of CpAcp and even peptidoglycan hydrolases, which will help to develop new antibiotics for combating multidrug-resistant bacteria.

## Figures and Tables

**Table 1:** Structural statistics for CpAcp SH3b6 ^1^.

Conformationally Restricting Constraints ^2^	
*NOE-based distance constraints*	
Total	822
Intra-residue (i = j)	168
Sequential (|i − j| = 1)	222
Medium-range (1< |i − j| <5)	69
Long-range (|i − j| ≥ 5)	363
*Hydrogen bond constraints*	
Long-range (|i − j| ≥ 5)/total	46/54
*Dihedral angle constraints*	50
**Residue Constraint Violations ^2^**	
*Average number of distance violations per structure*	
0.1–0.2 Å	3.05
0.2–0.5 Å	0.35
>0.5 Å	0
Average RMS distance violation/constraint (Å)	0.01
Maximum distance violation (Å)	0.36
*Average number of dihedral angle violations per structure*	
1–10°	0.25
>10°	0
Average RMS dihedral angle violation/constraint (degree)	0.12
Maximum dihedral angle violation (degree)	1.90
*RMSD from average coordinates ^2,3^*	
Backbone/heavy atoms (Å)	0.3/0.9
**Structure Quality Factors**	
*Ramachandran plot statistics ^2,3^*	
Most favored/allowed regions (%)	98.0/2.0
Disallowed regions (%)	0.0
*Global quality scores(raw/Z-score) ^2^*	
Verify3D	0.47/0.16
Prosall	0.31/−1.41
Procheck(phi-psi) ^3^	−0.36/−1.10
Procheck(all) ^3^	−0.13/−0.77
Molprobity clash	17.01/−1.39
*RPF Scores ^4^*	
Recall/precision	0.999/0.943
F-measure/DP-score	0.971/0.875

^1^ Structural statistics were computed for the ensemble of 20 deposited structures. ^2^ Calculated using the PSVS 1.4 program. Residues (469–535) were analyzed. ^3^ Ordered residues ranges (with the sum of φ and ψ order parameters > 1.8): 470–475, 480–484, 492–496, 498–508, 511–516, and 520–529. ^4^ RPF scores reflected the goodness-of-fit of the final ensemble of structures, including disordered residues, to the NMR data.

**Table 2:** Parameters of ligand-binding pockets of CpAcp SH3b6.

No.	Volume (Å^3^)	Surface (Å^2^)	Hydrogen Bond Donors/Acceptors	Hydrophobic Interactions	Amino Acid Ratio			
					Nonpolar	Polar	Positive	Negative
1	317.38	738.87	19/36	28	0.38	0.47	0.10	0.05
2	210.82	372.21	4/10	19	0.36	0.37	0.18	0.09
3	125.25	372.62	4/14	17	0.44	0.22	0.11	0.22

**Table 3:** Structure similarity analysis of CpAcp SH3b6 using Dali server. The proteins are ranked according to the structural similarity score (Z-score).

PDB	Uniprot	Z-Score	RMSD	Ident%	Organism	Description	Ref.
4KRT	Q0SPG7	8.6	1.8	27	Clostridium phage phiSM101	Endolysin, Psm	[21]
3PVQ	Q8A860	8.3	2.1	19	Bacteroides thetaiotaomicron	NlpC/P60 family, YkfC	[26]
3NPF	A7LS31	8.2	2.8	17	Bacteroides ovatus	NlpC/P60 family, YkfC	[26]
6BIQ	A2DC48	8.0	2.8	11	Trichomonas vaginalis	NlpC/P60 family, NlpC_A2	[27]
4Q2W	P59205	8.0	3.5	16	Streptococcus pneumoniae	N-acetylglucosaminidase, LytB	[14]
6BIM	A2D7D7	7.7	3.8	11	Trichomonas vaginalis	NlpC/P60 family, NlpC_A1	[27]
1R77	O05156	7.5	1.6	15	Staphylococcus capitis	Lysostaphin, ALE-1	[10]
5LEO	P10547	7.5	1.5	15	Staphylococcus simulans	Lysostaphin	[12]
6RK4	P10547	7.5	1.5	15	Staphylococcus simulans	Lysostaphin	[17]
6ILU	A0A218KCJ1	7.4	1.9	15	Bacillus phage PBC5	Endolysin, LysPBC5	[23]
6RJE	P10547	7.4	1.5	15	Staphylococcus simulans	Lysostaphin	[17]
5NMY	P10547	7.2	1.6	15	Staphylococcus simulans	Lysostaphin	[28]
2MK5	D6QY02	6.9	1.9	10	Staphylococcus phage G15	Endolysin, LysGH15	[29]
3H41	Q736M3	6.7	5.0	13	Bacillus cereus	NlpC/P60 family, YkfC	[22]

**Table 4:** Comparison of pocket size of SH3b1/2/3/6/10 of CpAcp.

No.	SH3b6			SH3b1			SH3b2			SH3b3			SH3b10		
	1	2	3	1	2	3	1	2	3	1	2	3	1	2	3
Volume (Å^3^)	317.4	210.8	125.3	204.7	102.5	166.1	147.6	121.9	167.9	208.6	142.1	116.2	160.5	180.7	135.2
Surface (Å^2^)	738.9	372.2	372.6	387.6	231.6	296.2	322.6	309.9	440.5	376.5	312.4	362.3	321.3	361.9	320.9

## Data Availability

Data supporting the reported results will be available from the corresponding author (Jiang Zhu).

## Sample Availability

Samples of the compounds are not available from the authors.
